# Mast Cell Activation, Neuroinflammation, and Tight Junction Protein Derangement in Acute Traumatic Brain Injury

**DOI:** 10.1155/2020/4243953

**Published:** 2020-06-24

**Authors:** Duraisamy Kempuraj, Mohammad Ejaz Ahmed, Govindhasamy Pushpavathi Selvakumar, Ramasamy Thangavel, Sudhanshu P. Raikwar, Smita A. Zaheer, Shankar S. Iyer, Casey Burton, Donald James, Asgar Zaheer

**Affiliations:** ^1^Department of Neurology, School of Medicine, University of Missouri, Columbia, MO, USA; ^2^Center for Translational Neuroscience, School of Medicine, University of Missouri, Columbia, MO, USA; ^3^Harry S. Truman Memorial Veterans Hospital, U.S. Department of Veterans Affairs, Columbia, MO, USA; ^4^Phelps Health, Rolla, MO, USA

## Abstract

Traumatic brain injury (TBI) is one of the major health problems worldwide that causes death or permanent disability through primary and secondary damages in the brain. TBI causes primary brain damage and activates glial cells and immune and inflammatory cells, including mast cells in the brain associated with neuroinflammatory responses that cause secondary brain damage. Though the survival rate and the neurological deficiencies have shown significant improvement in many TBI patients with newer therapeutic options, the underlying pathophysiology of TBI-mediated neuroinflammation, neurodegeneration, and cognitive dysfunctions is understudied. In this study, we analyzed mast cells and neuroinflammation in weight drop-induced TBI. We analyzed mast cell activation by toluidine blue staining, serum chemokine C-C motif ligand 2 (CCL2) level by enzyme-linked immunosorbent assay (ELISA), and proteinase-activated receptor-2 (PAR-2), a mast cell and inflammation-associated protein, vascular endothelial growth factor receptor 2 (VEGFR2), and blood-brain barrier tight junction-associated claudin 5 and Zonula occludens-1 (ZO-1) protein expression in the brains of TBI mice. Mast cell activation and its numbers increased in the brains of 24 h and 72 h TBI when compared with sham control brains without TBI. Mouse brains after TBI show increased CCL2, PAR-2, and VEGFR2 expression and derangement of claudin 5 and ZO-1 expression as compared with sham control brains. TBI can cause mast cell activation, neuroinflammation, and derangement of tight junction proteins associated with increased BBB permeability. We suggest that inhibition of mast cell activation can suppress neuroimmune responses and glial cell activation-associated neuroinflammation and neurodegeneration in TBI.

## 1. Introduction

Acquired brain injury may be due to traumatic or nontraumatic injury. Traumatic brain injury (TBI) is “an alteration in brain function or other evidence of brain pathology caused by an external force (open/penetrating or closed/nonpenetrating) (Brain Injury Association of America, Vienna, VA, USA).” TBI may be due to falls, assaults, vehicle accidents, sports activity-related injuries, head trauma, gunshots, job-related injuries, child abuse, domestic violence, military activities including blasts, etc. Non-TBI may be due to stroke, diseases, seizure, tumors, toxins, metabolic diseases, hypoxia, drug overdose, etc. Approximately 1.7 million Americans report TBI every year in the USA, and about 3 million people are living with a life-long disability [1]. TBI leads to direct primary brain damage and indirect secondary brain damages. The secondary brain injury is due to the neuroimmune and inflammatory response to TBI [2]. TBI may disrupt normal functions of the brain and other organs [3]. The severity of a TBI pathogenesis may range from “mild” with a slight change in mental status or consciousness to “severe” with unconsciousness or amnesia after the brain injury. The acute and chronic effects of brain injuries vary significantly from person to person or in animal models of TBI [3, 4]. Therefore, no two TBIs are the same with reference to the neuroimmune response and disease severity. The severity of a brain injury is determined by several factors including cause, location in the brain, age, gender, and the extent of damage (Brain Injury Association of America, Vienna, VA) [5]. Although the current therapeutic options significantly improve the survival rate and the neurological deficiencies, the underlying molecular and immunological mechanism of TBI is still largely unknown [6, 7].

Mast cells are effector cells in the immune and inflammatory system that is implicated in neuroprotection as well as detrimental neurodegeneration, depending upon the type and site of activation [8–12]. Mast cells are generally seen in different regions of the brain, including the meninges, entorhinal cortex, choroid plexus, olfactory bulb, mesencephalon, thalamus, and hypothalamus [13]. Activated mast cells release prestored (preactivated) and newly generated multifunctional inflammatory molecules including cytokines, chemokines, and neurotoxic molecules that either directly induce inflammation and blood-brain barrier (BBB) breakdown and/or activate astrocytes, microglia, and neurons to release additional such inflammatory mediators that further promote neuronal death and cognitive dysfunction in neurodegenerative diseases [8, 14–16]. Several research reports indicate that mast cells are early responders following brain injury to release many mediators that induce neuroprotective process but later may cause brain injury-associated pathogenesis [5, 17]. Proteinase activated receptors (PARs) are significantly expressed in the peripheral system and the central nervous system (CNS) [18]. PAR-1, PAR-2, PAR-3, and PAR-4 subtypes are implicated in many inflammatory conditions. Neurons, microglia, astrocytes, oligodendrocytes, and endothelial cells express PARs in the brain [19, 20]. PAR-2 level is increased in the brains with neuroinflammatory and neurodegenerative disorders [21–23]. Tryptase (protease) released by degranulation of mast cells can activate mitogen-activated protein kinases (MAPKs), nuclear factor kappa-B (NF-*κ*B), protein kinase C (PKC), and Ca^++^ pathways that are implicated in the generation of proinflammatory cytokines and chemokines through the activation of PAR-2 in glial cells and neurons [23, 24]. In this study, we analyzed mast cell activation, PAR-2, chemokine C-C motif ligand 2 (CCL2), vascular endothelial growth factor receptor 2 (VEGFR2), and tight junction proteins claudin 5 and Zonula occludens-1 (ZO-1) expression in weight drop-induced TBI brains of mice. We report increased mast cell activation and upregulated CCL2, PAR-2, and VEGFR2 expression along with the reduced levels of claudin 5 and ZO-1 in mouse TBI brains.

## 2. Materials and Methods

### 2.1. Reagents

Claudin 5 monoclonal antibody (AC3C2), VEGF receptor 2 monoclonal antibody (B.309.4), ZO-1 monoclonal antibody, polyclonal Glial Fibrillary Acidic Protein (GFAP) antibody, Alexa Fluor 568 donkey anti-rabbit (H+L) antibody, 4′,6-diamidino-2-phenylindole, dihydrochloride (DAPI), tissue extraction reagent, Dulbecco's phosphate-buffered saline (DPBS), BCA protein assay kit (Pierce), and 1-step ultra TMB-ELISA solution were purchased from Thermo Fisher Scientific (Rockford, IL). Goat anti-mouse IgG FITCs antibody and toluidine blue O were obtained from Sigma-Aldrich (St. Louis, MO). Anti-NeuN antibody (rabbit polyclonal) neuronal marker was obtained from Abcam (Cambridge, MA). CCL2 DuoSet enzyme-linked immunosorbent assay (ELISA) kit was obtained from the R&D Systems (Minneapolis, MN).

### 2.2. Weight Drop-Induced Closed-Head Acute TBI Model and Experimental Procedures

Wild-type (C57BL/6) 8-week-old male mice were used to induce weight drop-associated closed-head acute TBI. In this model, the mouse was anesthetized using isoflurane, and the head was placed on a spongy surface to allow the head to move along due to the weight fall. Then, the skull was exposed on the top with an incision. Following this, a metal weight (35 g) was released to free fall from 80 cm above the head through a vertical tube (guided path) to induce TBI without craniotomy, as previously reported by others for closed-head injury weight drop model [25–28]. Then, the skin was sutured for the recovery. The body temperature of mice was maintained at 37°C during these procedures. The only skin incision was made in sham control mice without the weight drop procedure. Then, the mice were returned to the cage for free access to water and food. After 24 h (*n* = 6 mice) and 72 h (*n* = 6 mice) of TBI, these mice and sham control mice (*n* = 6 mice) were euthanized, and the blood samples were collected, and the brains were removed and processed for section cutting using a cryostat. Then, the serum was separated from the clot by centrifugation of tubes at 2000 g for 10 min in a refrigerated centrifuge and stored at -80°C until CCL2 assay by ELISA. Brain tissue sections (20 *μ*m) were prepared from these brains for the detection of mast cells by toluidine blue and the analysis of PAR-2, NeuN, VEGFR2, claudin 5, and ZO-1 expression by immunofluorescence staining, as we have reported previously [21, 29]. Maintenance of mice and the experiments were carried out “according to the recommendations in the G*uide for the Care and Use of Laboratory Animals* of the National Institutes of Health (NIH) and the approval of the committee on the Ethics of Animal Experiments of the University of Missouri (Columbia, MO).”

### 2.3. Weight Drop-Induced Lesion

Cryostat brain sections (20 *μ*m) from TBI and sham control mouse brains were stained with 0.1% crystal violet staining solution for about 5 min, washed, processed in alcohol, cleared with xylene, and mounted. These sections were observed under a bright-field microscope using 20x objectives. Then, the images were captured to examine the lesion area.

### 2.4. Mast Cell Staining with 0.1% Toluidine Blue Stain

Cryostat sections from 24 h and 72 h TBI mice as well as from wild-type sham control mice without TBI were used to detect mast cells (*n* = 6 mice/group). Mast cells were detected in these brain sections after staining with 0.1% toluidine blue for 2 min. Mast cells were detected in these sections by violet color or different levels of blue color, as observed under the microscope, as we have reported previously [21]. The number of mast cells counted per field in the images under high-power magnification was provided in the graph. Mast cell number and their degranulation status were determined by their abnormal morphology and the presence of extracellular cytoplasmic granules under a bright-field microscope using 100x objective.

### 2.5. Quantification of CCL2 in the Brain and Serum of Acute TBI Mice

Brain tissue lysates were prepared using tissue extraction reagent and tearor. Protein concentration in the brain lysates was quantified using the BCA protein assay kit as per the directions of the manufacturer. CCL2 level was quantified in the brain lysate and serum using a commercial DuoSet ELISA kit as per the manufacturers' guidelines. The optical density of the plate was read at 450 nm with a microplate reader (Molecular Devices, Sunnyvale, CA), as we have reported previously [29–31].

### 2.6. Immunofluorescence Detection of PAR-2, NeuN, VEGFR2, Claudin 5, and ZO-1 in Acute TBI Brains in Mice

Cryostat sections from 24 h and 72 h TBI brains and sham control mouse brains were fixed with a 4% paraformaldehyde solution. Immunofluorescence labeling was performed using an anti-PAR-2 mouse monoclonal antibody (1 : 100) along with an anti-NeuN rabbit polyclonal antibody (1 : 500), as we reported [21, 29]. Briefly, the brain sections were incubated with mixed antibodies at 4°C overnight with gentle shaking and incubated with a mixture of Alexa Flour 488 goat anti-rabbit IgG (1 : 300) and anti-mouse IgG/goat anti-mouse Texas red secondary antibodies (1 : 500) for one hour at room temperature with gentle shaking for immunofluorescence labeling. Cellular nuclei were stained with DAPI. Then, the sections were washed with DPBS, mounted, dried, and viewed with a confocal fluorescent microscope (Leica Microsystems GmbH, Germany; at Harry S. Truman Memorial Veterans Hospital, Columbia, MO). Photomicrographs were taken using an oil immersion objective (40x or 63x), as we already reported [29, 32–36]. Similarly, we have performed double immunofluorescence staining for VEGFR2 (1 : 200), claudin 5 (1 : 100), and ZO-1 (5 *μ*g/ml) in the brain sections from 24 h and 72 h TBI brains as well as sham control mouse brains along with DAPI for cellular nuclei. The intensity of immunoreactivity was quantified in the images at three different fields using ImageJ software (National Institutes of Health, Bethesda, MD). The results were provided as % of sham control in the bar graphs [37].

### 2.7. Behavioral Study

#### 2.7.1. Novel Object Recognition (NOR) Test for Cognitive Performance

This test is used to assess visual memory function and object recognition memory in animal models of brain disorders, including TBI. NOR is based on the fact that rodents explore more with a novel object as compared to a familiar object. Typically, animals spend more time with novel objects than familiar objects. However, the mouse with cognitive impairment may not recognize familiar objects and thus may not spend more time with novel objects. In this test, first, the mouse was exposed to two similar objects (A, A) for 5 min a day before the TBI procedure to familiarize in an open field arena square plexiglass apparatus with high walls. The time spent near each object was recorded. Then, the mouse was removed and placed in the original cage. Next, the mouse is exposed to one familiar object (A) and one novel object (B) in the same site during familiarization training after 24 h and 72 h of TBI. Again, the amount of time spent near each object was recorded and determined the cognitive status, as reported previously in TBI [38, 39]. The plexiglass apparatus was cleaned with 70% ethanol between tests with each mouse.

### 2.8. Statistical Analysis

The results obtained from mast cell count, ELISA, and immunoreactivity intensity were statistically analyzed by one-way analysis of variance (ANOVA) and Tukey-Kramer multiple comparison analysis using GraphPad InStat 3 software. Significant differences between the sham control and TBI groups were determined. Results were provided as mean ± SEM.

## 3. Results

### 3.1. TBI-Induced Lesion and Activation of Astrocytes

**Figure 1:**
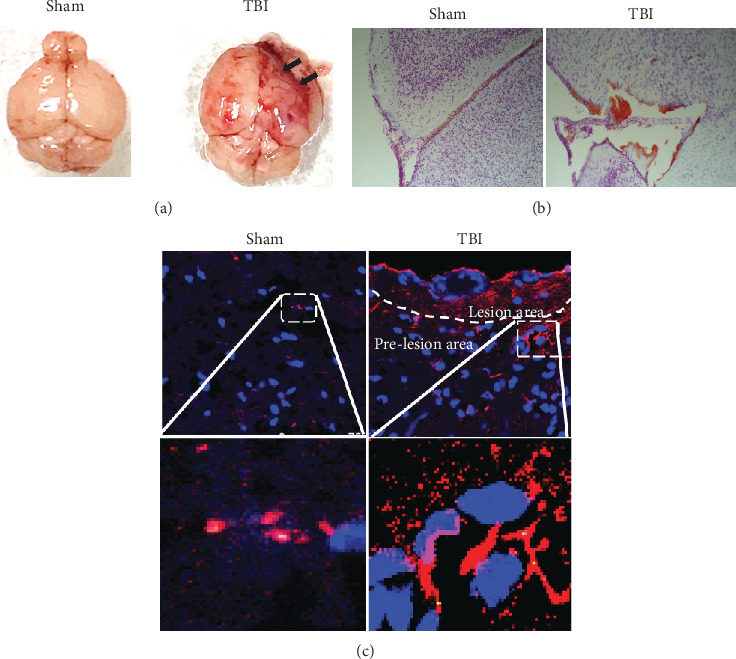
Closed-head weight drop-induced neuroinflammation. Representative images of (a) whole brains and 0.1% crystal violet staining indicate hemorrhage/inflammatory changes (b) in TBI brains as compared to sham control mouse brains (*n* = 6 mice/group). Representative microphotographs show increased GFAP immunoreactivity indicating an increased number of activated astrocytes in TBI mice (72 h) as compared to sham control mice (c). Image magnifications: (b) 200x and (c) 630x.

To confirm the effect of weight-induced TBI, the brains form sham control and TBI mice were removed and examined (*n* = 6). Representative image of whole brains (Figure 1(a)) and 0.1% crystal violet staining shows hemorrhage/inflammatory reactions in TBI brains (Figure 1(b)) as compared to sham control mouse brains. Representative microphotograph shows an increased number of astrocyte activation (red color) as determined by GFAP immunostaining for astrocytes in TBI mice (72 h) as compared to sham control mice (Figure 1(c)). These results show weight drop-induced astrocyte activation in the lesion area in the TBI mouse brain.

### 3.2. Acute TBI Increases Mast Cell Number and Its Activation in the Brain

**Figure 2:**
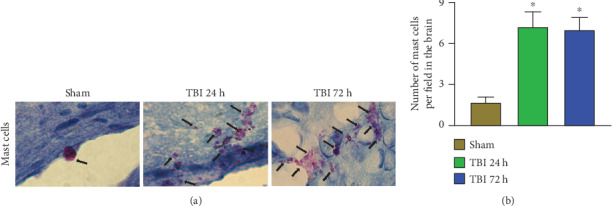
Acute TBI increases mast cell number and degranulation in the brain. Mast cell number and its degranulation were evaluated in the frozen sections (20 *μ*m) cut from the brains after 24 h and 72 h of weight drop-induced TBI and sham control mouse brains without TBI (*n* = 6 mice/group). These brain sections were stained with 0.1% toluidine blue solution for mast cell detection. The number, as well as activation (purple, black arrows) of mast cells, was increased in 24 h and 72 h TBI brains as compared with sham control mouse brains without TBI (a). Note the presence of widespread extracellular granules in degranulated mast cells. Degranulated mast cells also appear irregular in shape. Control mast cells without degranulation did not show extracellular cytoplasmic granules. Photomicrograph original magnifications = 100x. Representative photomicrographs show increased number and activation of mast cells in 24 h and 72 h TBI brains as compared with sham control brains without TBI (a). Photomicrograph original magnifications = 100x. The total number of mast cells was increased in TBI brains as compared to sham control mouse brains (b; ∗*p* < 0.05, sham control vs. TBI).

Mast cells were evaluated in the brain sections from 24 h and 72 h after weight drop-induced TBI and sham control mouse brains without TBI (*n* = 6 mice/group). These brain sections were incubated with 0.1% toluidine blue for mast cell detection in the brain. The number and the activation (purple, black arrows) status of mast cells were increased in 24 h and 72 h TBI brains as compared with sham control mouse brains without TBI (Figure 2(a)). Sham control mouse brains did not show mast cell degranulation. However, mast cells in the TBI brain show increased numbers as well as degranulation, as evidenced by the presence of extracellular widespread cytoplasmic granules (arrows). The degranulated mast cells show irregular shapes (arrows), unlike normal mast cells. The total number of toluidine blue-stained mast cells were increased in TBI brains when compared with sham control mouse brains (Figure 2(b); *p* < 0.05).

### 3.3. Increased Level of CCL2 in the Brain and Serum of Acute TBI

**Figure 3:**
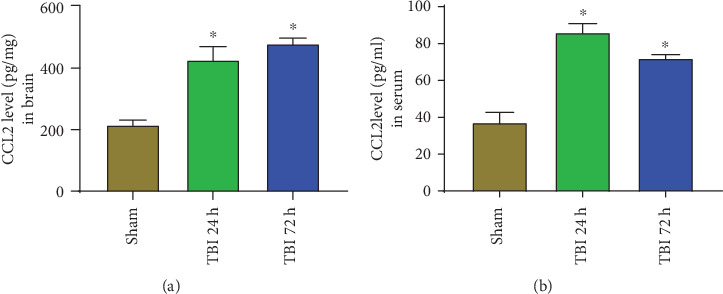
Increased level of CCL2 in the brain and serum of acute TBI mice. CCL2 level was quantified by ELISA in the brain tissue lysate and serum of 24 and 72 h acute TBI mice and sham control mice without TBI (*n* = 3). Results show significantly increased level of CCL2 in the (a) brains and (b) sera of both 24 h and 72 h acute TBI as compared with sham control mice (∗*p* < 0.05, sham control vs. TBI).

Next, we quantified CCL2 levels in the brain tissue lysates and sera from mice after 24 h and 72 h of weight drop-induced TBI (*n* = 3). Results show significantly increased brain and serum levels of CCL2 at both 24 h and 72 h after TBI as compared with the level in the sham control mice without TBI (Figures 3(a) and 3(b), *p* < 0.05). These observations indicate that the chemokine CCL2 level increases soon after brain injury.

### 3.4. Acute TBI Increases PAR-2 Expression in the Brain

**Figure 4:**
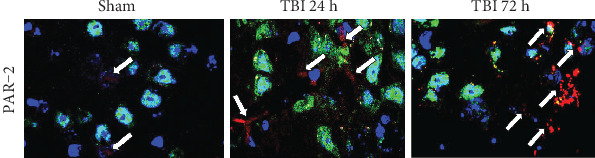
Acute TBI increases PAR-2 expression in the brain. PAR-2 expression was analyzed in the frozen sections (20 *μ*m) from the brains of 24 h and 72 h weight drop model of TBI and sham control mouse brains without TBI (*n* = 3 mice/group). We analyzed the expression of PAR-2 (red color) for inflammation and NeuN (green color) for neurons in these brain sections by triple immunofluorescence staining. Cellular nuclei were stained with DAPI (blue color). Representative photomicrographs show increased PAR-2 expression (red color, white arrows) in 24 h and 72 h acute TBI brains as compared with sham control mouse brains without TBI. Photomicrograph original magnifications = 630x.

PAR-2 expression was analyzed in the brain sections from 24 h and 72 h weight drop model of TBI and sham control mouse brains without TBI (*n* = 3 mice/group). We analyzed the expression of PAR-2 for inflammation, NeuN for neurons, and DAPI for cellular nuclei in these brain sections by immunofluorescence staining. Results show an increased level of PAR-2 expression (red color, white arrows) in 24 h and 72 h TBI brains as compared with sham control mouse brains without TBI (Figure 4). Cellular nuclei are shown in blue color (DAPI). Brain sections from 72 h after TBI show more PAR-2 expression as compared to 24 h brain sections.

### 3.5. Acute TBI Increases VEGFR2 Expression in the Brain

**Figure 5:**
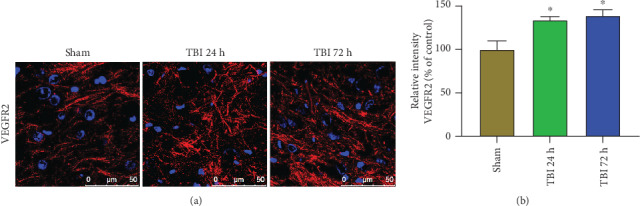
Acute TBI increases VEGFR2 expression in the brain. VEGFR2 expression was analyzed in the frozen sections (20 *μ*m) cut from the brains after 24 h and 72 h of weight drop model of TBI and sham control mouse brains without TBI (*n* = 3 mice/group) by immunofluorescence staining. Representative images and immunoreactivity intensity bar graphs show increased VEGFR2 expression (red color) in 24 h and 72 h acute TBI brains as compared with sham control brains without TBI (∗*p* < 0.05, sham control vs. TBI). The nuclei were stained with DAPI (blue color). Photomicrograph original magnifications = 630x.

VEGFR2 expression was analyzed in the sections from brains after 24 h and 72 h of weight drop-induced TBI and sham control mouse brains without TBI (*n* = 3 mice/group) by immunofluorescence staining. Immunoreactivity photomicrographs and staining intensity bar graphs show increased VEGFR2 expression (Figure 5(a); red color) in 24 h and 72 h acute TBI brains as compared with sham control mouse brains without TBI (Figure 5(b); *p* < 0.05). Cellular nuclei are shown in blue color with DAPI.

### 3.6. Acute TBI Affects Claudin 5 Expression in the Brain

**Figure 6:**
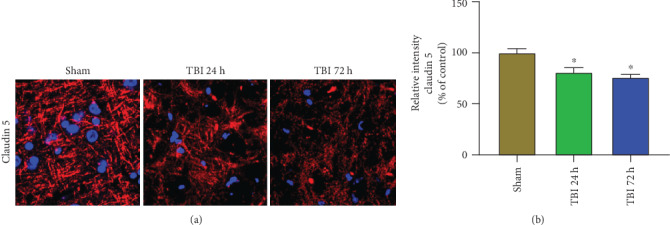
Acute TBI affects claudin 5 expression in the brain. Claudin 5 expression was analyzed in the frozen sections (20 *μ*m) of the brains after 24 h and 72 h of weight drop-induced TBI and sham control mouse brains without TBI (*n* = 3 mice/group) by immunofluorescent staining. Representative images and immunoreactivity intensity bar graphs show decreased/derangement of claudin 5 (red color, white arrows) expression in 24 h and 72 h acute TBI brains as compared with sham control mouse brains without TBI (∗*p* < 0.05, sham control vs. TBI). The nuclei were stained with DAPI (blue color). Photomicrograph original magnifications = 630x.

Tight junction-associated claudin 5 expression was analyzed in the brains after 24 h and 72 h of the weight drop model of acute TBI and sham control mouse brains without TBI (*n* = 3 mice/group) by immunofluorescence staining. Claudin 5 expression (red color) shows derangement and decreases in 24 h and 72 h TBI brains as compared with sham control brains without TBI (Figures 6(a) and 6(b); *p* < 0.05). Cellular nuclei are shown in blue color with DAPI.

### 3.7. Acute TBI Affects ZO-1 Expression in the Brain

**Figure 7:**
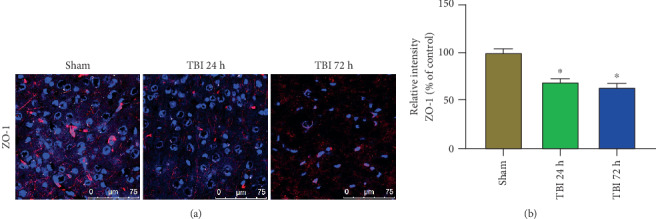
Acute TBI affects ZO-1 expression in the brain. ZO-1 expression was analyzed in the frozen sections (20 *μ*m) of the brains after 24 h and 72 h of weight drop-induced TBI and sham control mouse brains without TBI (*n* = 3 mice/group). Representative images and immunoreactivity intensity bar graphs show the derangement of ZO-1 expression (red color) in 24 h and 72 h acute TBI brains as compared with sham control brains without TBI (∗*p* < 0.05, sham control vs. TBI). The cellular nuclei were stained with DAPI (blue color). Photomicrograph original magnifications = 400x.

Tight junction-associated ZO-1 expression was analyzed in the brain sections after 24 h and 72 h of the weight drop model of acute TBI and sham control mouse brains without TBI (*n* = 3 mice/group). ZO-1 expression (Figure 7(a); red color) shows derangement and decrease in 24 h and 72 h acute TBI brains as compared with sham control mouse brains without TBI (Figure 7(b); *p* < 0.05). Cellular nuclei are shown in blue color with DAPI.

### 3.8. TBI Induces Cognitive Deficits as Assessed by NOR Test

**Figure 8:**
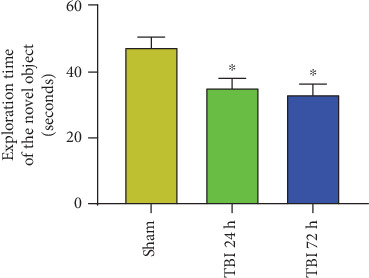
NOR test was conducted to assess memory function in TBI mice. The mouse was exposed to two similar objects (A, A) to familiarize in an open field arena box apparatus for 5 min a day before TBI procedures (*n* = 6 mice/group). The time spent near the objects was recorded. Then, the mouse was exposed to one familiar object (A) and a novel object (B) for 5 min, and the time spent at each object was recorded after 24 h and 72 h of TBI procedure. Results show that sham control mice show increased time spent at the novel object than at the familiar object (∗*p* < 0.05). However, TBI mice show poor performance as they did not recognize the familiar object, and the time spent at the familiar object and novel object did not show any significant variation.

NOR test was carried out a day before TBI induction, 24 h, and 72 h after TBI induction (*n* = 6 mice/group). NOR results show that the sham control mice show more time spent near the novel objects than the familiar objects indicating that sham control mice recognize the familiar objects and thus spent more time at the novel object. However, TBI mice show poor performance as they did not show increased time spent near the novel object since these mice did not recognize the novel object indicating the cognitive function is impaired (Figure 8).

## 4. Discussion

TBI is a significant health concern worldwide due to its morbidity and mortality. However, the exact mechanism of neuroimmune response after TBI is understudied. Therefore, we have investigated mast cell activation and expression of PAR-2, VEGFR2, claudin 5, and ZO-1 in the brains of mice after closed-head weight drop-induced acute TBI. We also measured CCL2 in the brain and serum of sham control and TBI mice in the present study. We demonstrate increased number and the degranulation of mast cells in 24 h as well as 72 h acute TBI brains as compared with sham control brains without TBI. Additionally, we detected the activation of meningeal (pia mater) mast cells after TBI. Further, acute TBI increased the levels of CCL2, PAR-2, and VEGFR2 and derangement of claudin 5 and ZO-1 expression in the brains as compared with sham control mouse brains. Weight drop-induced TBI was selected to simulate road accidents, falls, concussive head trauma, and domestic violence. Mast cells can promote brain damage by increasing BBB permeability, brain edema, extravasation, and hemorrhage in stroke by amplifying the neuroimmune response after mast cell activation [13]. The weight drop model is widely used as an animal model of TBI. However, the histopathological changes, immune and inflammatory responses, and behavioral changes vary, as there are many variations in the experimental procedures and animals used [1, 27]. The mechanism and the nature of the neuroimmune response, including mast cell response, are more complex to understand after neurotrauma/TBI. A better understanding of the mechanisms involved in TBI-mediated neuroimmune and mast cell responses is essential to develop novel approaches and an active therapeutic agent for TBI. However, rodent and human mast cells vary in phenotype, the immune response to stimuli, and the spectrum of mediators (pro- and anti-inflammatory) released. Thus, the findings with mast cells in rodents should also be evaluated in patients, as reported previously [13]. Mature mast cells can move from the periphery into the brain under different pathophysiological conditions [13].

A recent report shows that meningeal mast cells are essential effector cells in the pathogenesis of stroke [40]. This is because all the blood vessels pass through the meninges before going into the brain. This makes the meninges and the resident immune cells act as a protective gatekeeper to battle for the brain parenchyma. Meningeal mast cells are long-term resident immune cells that are filled with preformed and preactivated mediators stored in the electron-dense cytoplasmic secretory granules and are ready to be released by degranulation process within a few seconds when needed. Thus, these meningeal resident mast cells act as readily armed soldiers at the meningeal gate [41]. Thus, meninges play a significant role in brain-immune interaction in various brain disorders, including brain injury and stroke [40]. Dura mater contains more resident mast cells than meningeal membranes. We did not obtain dura in this study to analyze mast cells. However, our future studies will focus on dura mater and other meningeal membranes in TBI. Further, cerebral mast cells were shown to increase BBB permeability in stroke [42]. Mast cells can provide neuroprotection initially, as mast cell-derived heparin and proteases may help to dissolve the blood clot after a stroke [40, 43]. Mast cells are considered as the “first responders” after an injury in the brain to protect it [10, 44–46]. Mast cell degranulation products such as histamine, TNF-*α*, heparin, transforming growth factor-beta (TGF-*β*), and proteases released immediately after an injury can provide neuroprotection and perform wound healing. Mast cells are essential in the wound healing process, including infected wounds and tissue repair mechanisms in the body [47, 48]. Over and sustained mast cell activation can be deleterious and neurotoxic based upon the tissue and disease conditions [49]. Mast cell activation-mediated histamine and other mediators contribute to the common posttraumatic headache in TBI patients [49]. About 18-58% of TBI patients show posttraumatic headache (PTH) for one year after trauma, and PTH is a significant severity predictor after concussion [50–52]. Acute activation of meningeal mast cells contributes to chronic pain and targeting mast cells for early prophylactic treatment after mild closed-head injury [53]. Our results show that the mast cell number and their activation status increased in TBI brains. However, it should be noted that the number of mast cells, activation of mast cells, and mast cell response differ between mice and humans. We detected mouse mast cells in the brain using toluidine blue staining as it is commonly used. Moreover, there is no single tryptase in the mouse to perform immunostaining to detect all the mast cells. Human mast cells are broadly classified into two types (MCT type containing tryptase and MCTC type containing both tryptase and chymase) but not in the mouse. Mouse mast cells are different and many types based upon the type of mouse mast cell proteases and thus difficult to identify specific types. So, we did not examine specific types of mast cells after TBI in this study. We and others have previously reported that in a physiological condition, the total number of mast cells present in the brain is limited. However, mast cells are powerful cells, and even a few mast cells can release sufficient quantities of inflammatory mediators that can affect the blood-brain barrier and activate glia and neurons in the brain [54, 55].

Glial cells and neurons express CCL2 in the brain that can recruit immunocytes into the site of inflammation for the neuroprotective function to limit brain damage [56]. However, high levels of CCL2 can cause extensive infiltration of immune cells, increase BBB permeability, and promote neuroinflammation and edema [57, 58]. Thus, we assayed CCL2 in this study. TBI and intracerebral hemorrhage patients show elevated plasma and serum levels of CCl2 as early as 2 h onwards and show poor outcomes [59, 60]. Our present study has shown increased CCL2 levels in the brain and the serum of weight drop-induced TBI mice. Increased CCL2 in TBI may induce the recruitment of immune cells, including mast cells and its activation-associated neuroinflammatory pathways. Neuroinflammatory conditions show increased PAR-2 expression [21–23]. Tryptase released from mast cells can increase the release of neuroinflammatory mediators from the glial cells [23, 24]. Mast cell proteases such as tryptase can increase PAR expression and upregulate the expression of several inflammatory cytokines and chemokines in neuroinflammation. The increased PAR-2 expression observed in this study in 24 h and 72 h TBI brains may be due to the increased mast cell activation. Thus, we suggest that inhibition of PAR-2 expression may be a new therapeutic target for the TBI, as previously suggested for neuroinflammatory conditions [20].

VEGF is a significant regulator of vascular permeability, including microvascular permeability in the brain. However, increased levels of VEGF and VEGFR2, the receptor for VEGF at the site of inflammation, can cause increased BBB permeability, edema in neuroinflammatory conditions including neurotrauma, and brain microvascular endothelial activation [61, 62]. VEGFR2 is known to be expressed by neurons, glia, and endothelial cells in the brain [63]. Brain injury induces angiogenesis in the brain. Marmarou's acceleration impact model and other brain injury models are known to increase VEGF and VEGFR2 expression [64, 65]. Our present study also reports increased VEGFR2 in TBI brains as compared to sham control mouse brains, indicating an immune response to TBI. Endothelial cells are structurally connected by several tight junction proteins that form BBB and regulate microvascular permeability in the brain. Claudin 5 and ZO-1 are the essential components of BBB that form tight junctions and regulate BBB permeability. Derangements or downregulation of these tight junction proteins can increase BBB permeability and edema in the brain. Increased intracranial pressure due to edema and inflammation can further cause tight junction protein downregulation and or derangements with BBB dysfunction and damage. Neurotrauma, including fluid percussion injury (FPI), can reduce tight junction proteins after 24 h of injury in mice [66]. These previous findings corroborate our present observation that claudin 5 and ZO-1 levels show derangement and downregulation in 24 h and 72 h TBI brains. Increased PAR-2 expression can cause derangements and downregulation of claudin 5 and ZO-1 expression, as also observed in this study. The presence of high levels of CCL2 in the sera in the present study indicates the disruption of BBB and leak out of CCL2 across the damaged BBB. Similarly, immunocytes, including mast cells, can move into the brain across the damaged BBB after TBI. Mast cell-derived inflammatory mediators can increase BBB permeability [67]. Moreover, a recent study has shown that CCL2 can induce tight junction protein disassembly at the endothelial cells in the brain [57]. The severity of TBI is more dependent on the edema/swelling due to increased BBB permeability than the primary injury itself.

**Figure 9:**
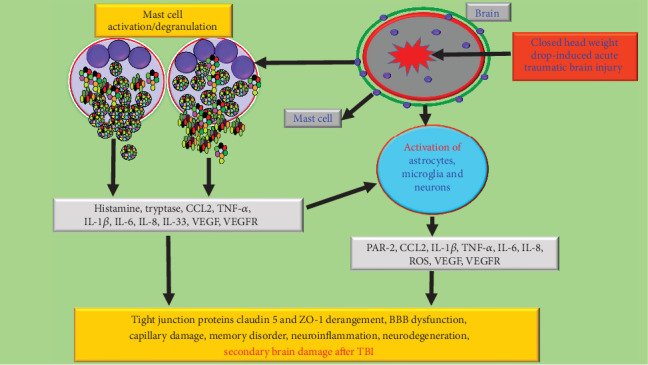
Schematic representation of mast cell activation in neuroinflammation, BBB disruption, and neuronal death in closed-head acute TBI brain. Neurotrauma/TBI can cause primary damage to neurovascular and gliovascular units in the brain with subsequent neuroimmune and neuroinflammatory responses. This leads to the activation of immune cells, including microglia, astrocytes, and mast cells in the brain. Mast cell activation causes degranulation and release of prestored and preactivated mediators such as histamine, proteases, and TNF-*α* followed by newly synthesized cytokines, chemokines, and neurotoxic mediators that can act on glial cells and neurons. Subsequently, peripheral immune as well as inflammatory cells can infiltrate the region of injury in the brain due to the BBB breach. CCL2 released from mast cells and brain cells induces the infiltration of inflammatory cells. Activated glial cells release neuroinflammatory mediators that further activate glial cells, neurons, and mast cells in a vicious fashion and induce PAR-2 and VEGFR2 expression and BBB breach with decreased tight junction protein such as claudin 5 and ZO-1 and its derangements. This continuous process can cause and upregulate neuroinflammation and neuronal death and secondary brain damage after TBI.

Neurotrauma or TBI produces direct primary damage to neurovascular and gliovascular units in the brain. This primary brain damage may lead to immediate neuroimmune and neuroinflammatory responses that result in the activation of immune cells, including microglia (resident immune cells), astrocytes, and mast cells in the brain parenchyma and resident mast cells in the meninges. Mast cell activation is associated with degranulation and release of prestored and preactivated histamine, heparin, and TNF-*α*, followed by newly synthesized cytokines, chemokines, and neurotoxic mediators that can act on microglia, astrocytes, and neurons. These activated glial cells and neurons release several neuroinflammatory mediators that can further act on glial cells, neurons, endothelial cells, pericytes, and mast cells in a vicious cycle. CCL2 released from mast cells and brain cells cause infiltration of immunocytes to the site of injury in the brain. This causes increased PAR-2 and VEGFR2 expression, BBB permeability, reduced tight junction protein, and its derangements and edema. This continuous vicious process can cause and upregulate neuroinflammation and neuronal death and secondary brain damage after TBI, as shown in Figure 9.

## 5. Conclusions

In this study, we have shown that mast cell activation can cause neuroinflammation and BBB disruption in weight drop-induced acute TBI brain. However, further in-depth ultrastructural and neuroimmunological studies are needed to better understand the precise mechanisms involved in the neuroprotective, neurodegenerative, and neurotoxic mechanisms after various types of TBI. Our future studies will focus on the quantification of mast cells in the brain and meningeal membranes, tight junction proteins, and other key mast cell and other neuroinflammatory biomarkers and therapeutic options for TBI.

## Data Availability

The data used to support the findings in the study are available on reasonable request to the corresponding authors.
